# Retraction: Down-regulation of XIAP enhances the radiosensitivity of esophageal cancer cells *in vivo* and *in vitro*

**DOI:** 10.1042/BSR-2017-0711_RET

**Published:** 2021-06-08

**Keywords:** Esophageal cancer, Gene silencing, Radiosensitivity, siRNA, XIAP

This Retraction follows an Expression of Concern relating to this article previously published by Portland Press.

This article is being retracted from *Bioscience Reports* at the request of the Editor-in-Chief and the Editorial Board following receipt of a notification from a reader, alerting the Editorial Board to the GAPDH Western blots in Figures 1C, 3B and 3E, and the tumor images in Figure 7C which are duplicated in several other publications the same author group, as well as by different authors.

The authors have been contacted in regards to the retraction, and have not responded to the Journal's queries and the concerns raised. Given the extent of the issues raised, the Editorial Board stand by the decision to retract the article.

